# Chemical composition of Polish propolis and its antiproliferative effect in combination with *Bacopa monnieri* on glioblastoma cell lines

**DOI:** 10.1038/s41598-020-78014-w

**Published:** 2020-12-03

**Authors:** Justyna Moskwa, Sylwia K. Naliwajko, Renata Markiewicz-Żukowska, Krystyna J. Gromkowska-Kępka, Patryk Nowakowski, Jakub W. Strawa, Maria H. Borawska, Michał Tomczyk, Katarzyna Socha

**Affiliations:** 1 Department of Bromatology, Faculty of Pharmacy with the Division of Laboratory Medicine, Medical University of Białystok, Mickiewicza 2D, 15-222 Białystok, Poland; 2 Department of Pharmacognosy, Faculty of Pharmacy with the Division of Laboratory Medicine, Medical University of Białystok, Mickiewicza 2a, 15-230 Białystok, Poland

**Keywords:** CNS cancer, Nutrition, Cancer, Plant sciences, Neurology, Chemistry

## Abstract

Propolis and *Bacopa monnieri* (L.) Wettst. (Brahmi) are natural products that contain many active substances and possess anticancer properties. The aim of this study was to investigate the chemical composition of Polish propolis extract (PPE) by gas chromatography-mass spectrometry (GC–MS), *B. monnieri* extracts (BcH, BcS) by high performance liquid chromatography with diode array detector and mass spectrometry coupled with electrospray ionization (LC–ESI–MS) and finally determine its anti-proliferative potential combined with BcH and BcS in glioblastoma cell lines (T98G, LN-18, U87MG). The antiproliferative activity of PPE, BcH, BcS and their combination (PPE + BcH) was determined by a cytotoxicity test, and DNA binding was determined by [^3^H]-thymidine incorporation. Flavonoids and phenylpropenoids were the main components of PPE. BcH and BcS samples were also successfully analyzed. Their main constituents were saponins such as bacoside A3, bacopaside II, X and bacopasaponin C and its isomer. The inhibitory effects on the viability and proliferation of the tested glioma cells observed after incubation with the combination of PPE and BcH were significantly stronger than the effects of these two extracts separately. These findings suggest that propolis in combination with *B. monnieri* shows promising anticancer activity for the treatment of glioblastoma. However, further studies are still required.

## Introduction

Glioblastoma multiforme (GBM) is a highly malignant brain tumor with an extremely poor prognosis. Standard therapies, including surgery, chemotherapy (temozolomide—TMZ), radiation therapy, hormonal therapy and targeted therapy, are frequently ineffective because numerous patients with GBM do not respond to TMZ as the tumor is resistant to treatment. The median survival time is only 14–15 months after diagnosis^1–3^. The data suggest that an increasing number of cancer patients use some form of complementary and alternative medicine (35.9%) to support therapy^4^. Therefore, there is a great need to understand the underlying mechanisms of tumor progression and search for new substances with anticancer potential.

Propolis is a mixture of tree and shrub resin, beeswax, pollen and secretions of bee glands with a characteristic balsamic smell and taste. The chemical composition of propolis depends on the vegetation of the geographical area it comes from, the season of the year and the breed of bees. Therefore, propolis shows different biological activities in studies by many authors. The most active compounds are flavonoids (e.g., chrysin, apigenin, pinocembrin, pinobanksin, kaempferol), aromatic acids (e.g., *p*-coumaric, ferulic) and esters (caffeic acid phenethyl ester, CAPE)^5,6^. Many researchers have confirmed the antioxidant, antimicrobial and anti-inflammatory activities of propolis^7,8^. The anticancer effects of propolis have been observed in many cancer cell lines, such as human lung cancer (A549), human prostate cancer (PC3), human myeloid leukemia (U937) and other cell lines^9–13^. In our previous study, we confirmed that propolis and other bee products have anti-proliferative potential in the human glioblastoma multiforme cell line U87MG^5,14,15^.

*Bacopa monnieri* (L.) Wettst., locally known as Brahmi, is a plant species from the Plantaginaceae family that naturally occurs in wetlands and swamps. *B. monnieri* is one of the most important plants in Ayurveda Chinese medicine. It has been used traditionally in the treatment of epilepsy and insomnia and as a sedative in anxiety disorder. The most important active compounds of this plant are triterpene saponins called bacosides^16,17^, alkaloids such as bramine and herpestine, flavonoid and steroid compounds^18,19^. In vivo studies have shown that *B. monnieri* extract containing 25% bacoside A causes an anxiolytic effect comparable to that of lorazepam. A frequent side effect of lorazepam is amnesia, whereas *B. monnieri* does not cause such side effects and additionally exhibits memory-improving properties^20^. Research has demonstrated that *B. monnieri* acts as an antioxidant and anti-inflammatory agent^21,22^ and increases the activity of superoxide dismutase (SOD), glutathione peroxidase (GPx) and catalase^23^. Furthermore, the authors confirmed its protective effect on the liver^24^ as well as its antimicrobial^25^ and antiulcer activity^26^. Recent research suggests that bacoside A has possible anticancer activity that may induce cell cycle arrest and apoptosis through the Notch signaling pathway in GBM in vitro^27^.

In the present study, the chemical composition of Polish propolis extract (PPE) was analyzed, and its anti-proliferative potential when combined with *B. monnieri* extracts from commercial formulations (BcH, BcS) was investigated in glioblastoma cell lines (T98G, LN-18, U87MG). This study provides new insights into the possible mechanism underlying the cytotoxic activity of PPE combined with *B. monnieri* in GBM cells.

## Results

### Chemical composition of propolis (PPE)

**Figure 1:**
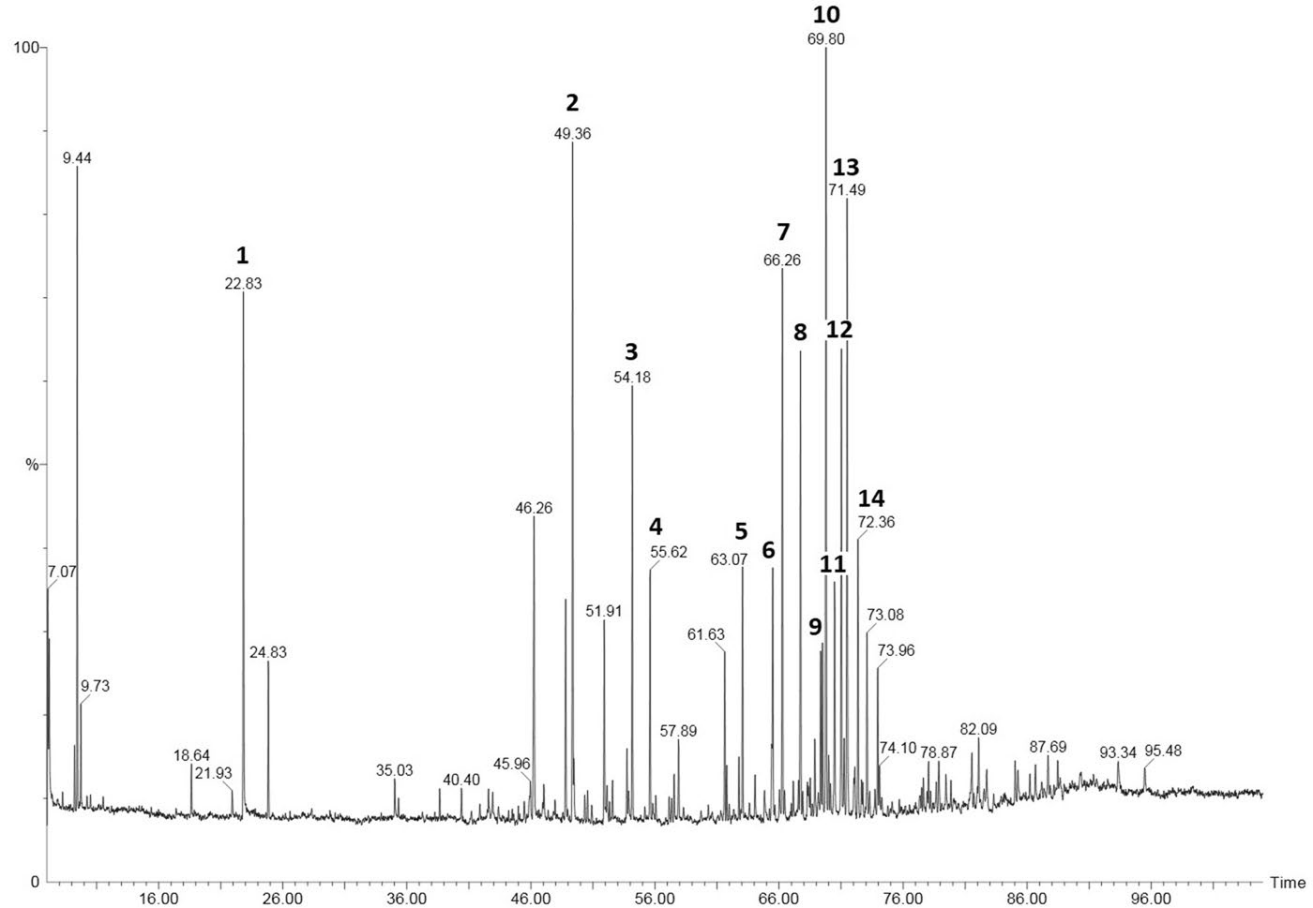
GC–MS of ethanol extract of propolis (PPE). Main components—1: Benzoic acid, 2: (E)-*p*-Coumaric acid, 3: Ferulic acid, 4: (E)-Caffeic acid, 5: 3-Methyl-2-butenyl-(E)-caffeate, 6: Benzyl *p*-Coumarate (E), 7: Pinocembrin, 8: Pinobanksin, 9: Benzyl-(E)-ferulate, 10: Pinobanksin 3-acetate, 11: Benzyl (E)-caffeate, 12: Chrysin, 13: Galangin, 14: CAPE.

In this study, 77 ingredients of PPE were identified. A list of these constituents is presented in Table 1 and Fig. 1. Flavonoids and chalcones (35.13%) were the main components of PPE. The major ingredients of this group were pinobanksin (13.0%), pinocembrin (5.7%), chrysin (5.4%), galangin (5.4%) and their derivatives. Phenylpropenoids, such as (E) *p*-coumaric acid (6.1%), ferulic acid (3.4%), benzyl-(E)-ferulate (3.3%), and benzyl *p*-coumarate-(E) (3.1%), were the second most abundant group (25.72%) Other compounds identified in PPE were present form various groups of constutuents, such as cinnamic acid esters, aliphatic acids, phenylpropenoids glycerides, aliphatic and aromatic alcohols, phenylpropenoids, sesquiterpenols, carbohydrates and other unidentified (NN) compounds.

**Table 1:** Chemical composition of the PPE.

Components, TMS derivative	Rt, min	LTPRI^Exp^	LTPRI^lit^	Relative composition (%)
Benzyl alcohol	18.66	1157	1156	0.5
2-Phenyl ethanol	21.95	1229	1227	0.4
Benzoic acid	22.85	1249	1248	4.8
Glycerol	24.83	1293	1293	1.3
Vaniline	35.05	1541	1542	0.7
Cinnamic acid	35.32	1547	1546	0.4
4-Hydroxybenzoic acid	38.63	1634	1636	0.4
6-Hydroxy-β-caryophyllene	40.41	1683	1682	0.6
n.n. (44, 73,281,75)	41.80	1721	–	0.2
n.n. (130, 73, 90,44)	41.89	1724	–	0.4
γ-Eudesmol	42.63	1745	1740	0.8
β-Eudesmol	42.93	1754	1751	0.6
Benzyl benzoate	43.40	1768	1765	0.3
(*Z*) *p*-Coumaric acid	44.55	1801	1798	0.2
n.n. (73,204,147,143)	45.49	1829	–	0.3
α-Fructofuranose	45.92	1842	1845	0.8
β-Fructofuranose	46.23	1852	1854	3.6
α-Mannofuranose	47.02	1876	1874	0.7
α-Glucopyranose	48.80	1930	1929	1.8
(*E*) *p*-Coumaric acid	49.39	1949	1949	6.1
n.n. (44, 73,281, 75)	50.35	1979	–	0.2
n.n. (130, 73, 90,44)	50.92	1997	–	0.2
β-Glucopyranose	51.93	2029	2032	1.8
3,4-Dimethoxycinnamic acid	52.13	2036	2034	0.5
Hexadecanoic acid	52.56	2051	2052	0.4
Isoferulic acid	53.80	2092	2088	1.1
Ferulic acid	54.18	2104	2104	3.4
(*E*)-Caffeic acid	55.60	2153	2156	2.4
2-Methyl-2-butenyl-(*E*)-p-coumarate	57.17	2207	2203	0.3
3-Methyl-2-butenyl (*E*)-p-coumarate	57.35	2214	2216	0.3
Oleic acid	57.56	2222	2222	0.6
n.n. (73, 147, 156, 233, 75)	57.89	2234	–	1.0
Stearic acid	58.30	2249	2249	0.3
2(3)-Methylbutanyl-(*E*)-caffeate	60.28	2353	2358	0.4
3-Methyl-3-butenyl-(*E*)-caffeate	61.61	2368	2367	1.8
2-Methyl-2-butenyl-(*E*)-caffeate	62.75	2414	2413	0.5
3-Methyl-2-butenyl-(E)-caffeate	63.07	2427	2424	2.1
Eicosanoic acid	63.63	2448	2447	0.2
Pinocembrin, mono-TMS	64.08	2466	2461	0.6
2′,6′,α-Trihydroxy-4′-methoxychalcone	64.87	2497	2492	0.5
Benzyl *p*-Coumarate-(*E*)	65.45	2520	2515	3.1
Pinocembrin chalcone	66.06	2544	2541	0.2
Pinocembrin	66.26	2551	2552	4.9
n.n. (192, 73, 297, 311)	67.17	2589	–	0.6
Pinobanksin	67.73	2606	2611	4.7
NN (73,297, 253)	68.34	2619	–	1.1
Chrysin, mono-TMS	68.90	2662	2655	0.9
Benzyl-(*E*)-ferulate	69.44	2685	2680	3.3
Pinobanksin 3-acetate	69.78	2700	2695	7.4
n.n. (73, 361, 217)	70.00	2709	–	0.8
Benzyl (*E*)-caffeate	70.47	2729	2723	2.2
Chrysin, di-TMS	71.01	2752	2746	4.5
5,7-Dihydroxy-3-methoxyflavanone, di-TMS	71.24	2762	2760	0.4
Galangin, tri-TMS	71.49	2773	2769	5.4
Pinobanksin 3-isobutanoate	72.07	2794	2791	0.7
2-Phenylethyl (*E*)-caffeate, CAPE	72.36	2805	2805	2.3
Isosakuranetin, 5,7-dihydroxy-4′-methoxyflavanone-TMS	72.70	2823	2820	0.6
Tetracosanoic acid	73.11	2845	2844	1.7
Pinobanksin 3-*n*-butanoate	73.29	2853	2849	0.2
Sakuranetin chalcone	73.74	2873	2870	0.3
Sakuranetin	73.96	2884	2880	2.2
Unidentified chalcone (280, 73)	74.77	2921	2918	0.1
n.n. (73, 44, 75, 189)	75.13	2937	–	0.4
n.n. (73, 300,147, 305)	75.72	2964	–	0.2
n.n. (44, 73, 207)	75.85	2975	–	0.1
n.n. (117, 115, 249, 73)	76.17	2985	–	0.2
Acacetin	76.55	2998	2991	0.2
n.n. (386, 73, 297,135)	76.98	3023	–	0.3
5,7,4′-Trihydroxy-3′-methoxyflavanone	77.59	3053	3050	0.6
n.n. (83, 413, 111)	78.01	3073	–	1.1
Kaempferol	78.89	3115	3114	0.8
6-Hydroxy-β-caryophyllene *p*-coumarate	79.48	3145	3136	0.7
n.n. (472, 73, 228, 474)	79.84	3163	–	0.4
14-Hydroxy-β-caryophyllene, *p*-coumarate	80.13	3177	3165	0.4
n.n. (73, 103, 301, 147)	81.52	3250	–	1.4
1,3-di-*p*-coumaroyl glycerol, tri-TMS	93.36	3865	3870	1.2
2-Acetyl-1,3-di-*p*-coumaroyl glycerol, TMS	95.50	3963	3960	1.0

n.n.—not identified; LTPRI*—*linear temperature-programmed retention index system.

### LC–ESI–MS analysis of *B. monnieri* extracts (BcH, BcS)

**Figure 2:**
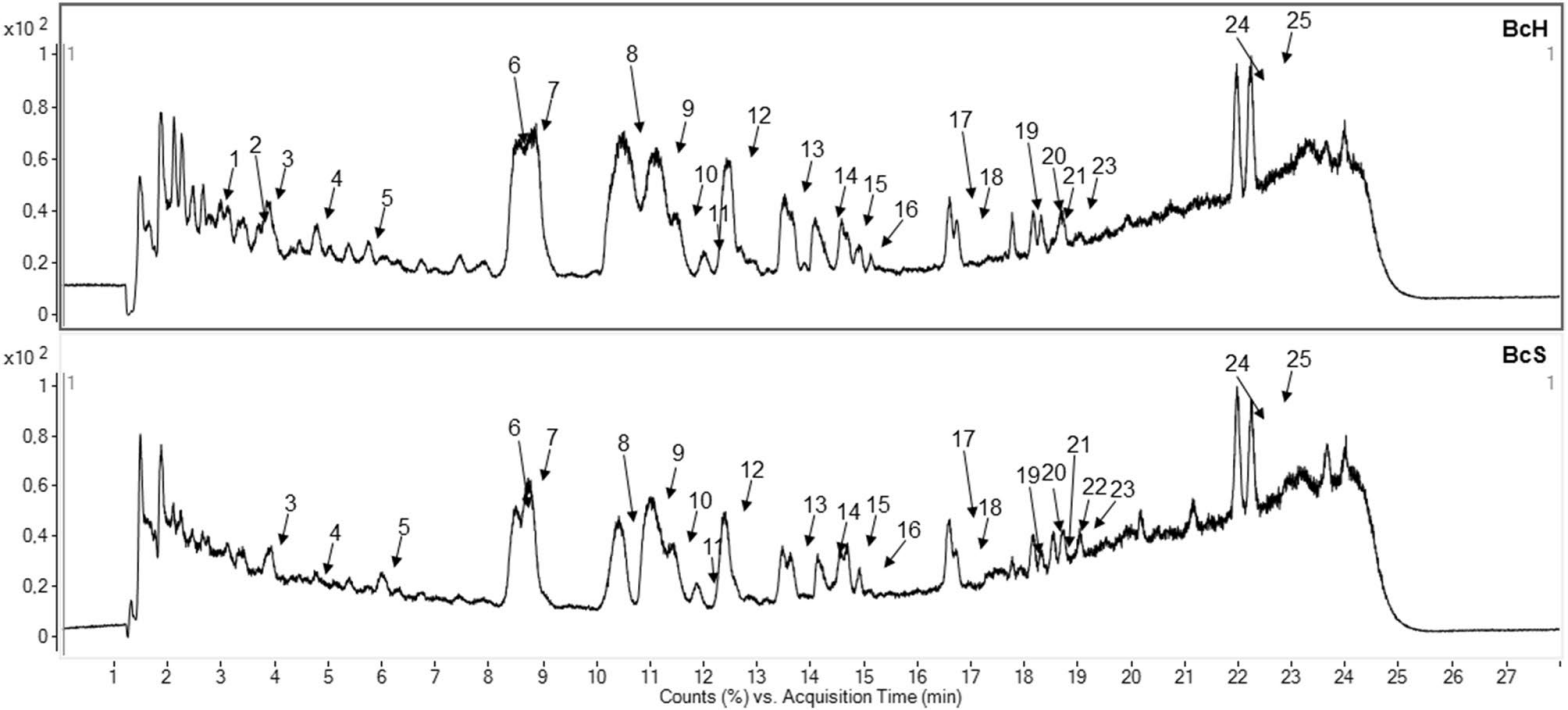
The qualitative assessment of commercial *Bacopa monnieri* L. extracts (BcH, BcS) using LC–ESI–MS in positive mode ionisation.

Among them 25 compounds were identified. Characteristic groups of compounds are both a complex of triterpene saponins—bacoside A3, A6, bacopaside II, IV, V, X, bacosaponin C with their isomers as well as a flavone of derivatives such as apigenin and luteolin. The results of the qualitative analysis of BcH and BcS are presented in Table 2 and Fig. 2.

**Table 2:** Qualitative LC–MS analysis of BcH and BcS.

No		Rt *min*		Negative mode *m/z*	Positive mode *m/z*	Tentatively identified compound
1		3.00		177, 257, **369**	-	Bacopaside A derivatives
2		3.81		1101, **1137**, 1147	**455**, 487, 617, 779, 911	j diglc-arab-acetylpentoside
3		3.96		133, **285**	**287**	Luteolin_(s)_
4		4.70		877, 1071, **1107**, 1117	**455**, 909, 1095	j diglc-arab-acetylpentoside
5		5.92		**269**	**271**	Apigenin_(s)_
6		8.49		795, 927, **963**, 973	**455**, 617, 779, 911,	Bacoside A3_(s)_
7		8.81		795, 927, **963**, 973	**455**, 617, 779, 911,	Bacopaside II_(s)_
8		10.53		897, **933**, 943, 993	**455**, 587, 749, 881, 921, 944	Bacopaside X_(s)_
9		11.15		897, **933**, 943	**473**, 605, 767, 921	Bacopasaponin C_(s)_
10		11.51		897, **933**, 943	473, 605, **767**	Bacopasaponin C isomer_(s)_
11		12.02		n.d	**274**	Imino diethanol
12		12.30		795, **831**, 1149	455, 617, 779, **797**	Unknown
13		13.53		765, **801**, 811	**455**, 587, 749	Bacopaside IV or V
14		14.12		**845**	473, 605, **767**	Bacopasaponin D
15		14.60		n.d	455, **635**	Unknown
16		14.81		963, **1059**, 1095	455, 605, **737**	Bacoside derivatives
17		16.61		n.d	473, **587**	Unknown
18		16.72		n.d	**605**	Unknown
19		17.76		**909**, 945	455, 617, **911**	Bacoside A6 isomer
20		18.15		**909**, 945	455, 617, **911**	Bacoside A6 isomer
21		18.23		453, 488**, 915**, 925	**881**	Deoxy-p-ara-glc-ara isomer
22		18.54		n.d	**445**, 490	Unknown
23		18.72		453, 488**, 915**, 925	**881**	Deoxy-p-ara-glc-ara isomer
24		21.97		n.d	386, **455**	Unknown
25		22.23		n.d	386, **473**	Unknown

j-jujubogenin; p-pseudojujubogenin ara-arabinose; glc-glucose; (s)—reference substance; bolded—peak with the highest abundance; n.d. not detected.

### Quantitative assessment of saponins in BcH and BcS

Bacoside A3 and Bacosaponin C were quantified using HPLC method in a homogenized sample of commercial formulation (BcS and BcH) from *B. monnieri*. BcS contains 7.92 ± 0.25 mg of bacoside A3 and 10.69 ± 0.12 mg of bacopasaponin C in 1 g of extract. BcH contains 27.77 ± 0.09 mg of bacoside A3 and 31.57 ± 0.07 mg of bacopasaponin C in 1 g of extract. The results of the quantitative analysis of BcH and BcS are presented in Table 3.

**Table 3:** Qualitative analysis of BcH and BcS.

Extract	Bacoside A3 (mg/g extract) Mean ± SD	Bacosaponin C (mg/g extract) Mean ± SD
BcH	27.77 ± 0.09	31.57 ± 0.07
BcS	7.92 ± 0.25	10.69 ± 0.12

### Total phenolic content (TPC)

The total phenolic content of PPE, BcH, and BcS was determined using the Folin–Ciocalteu assay and found to be 137.19 ± 3.96, 14.80 ± 0.18, and 14.57 ± 0.21 mg GAE/g, respectively (Table 4).

**Table 4:** Total phenolic content (TPC) of the PPE, BcH and BcS.

Extracts	TPC (mg GAE/g) Mean ± SD
PPE	137.19 ± 3.96
BcH	14.80 ± 0.18
BcS	14.57 ± 0.21

TPC—milligrams of gallic acid equivalent [GAE] per gram of dry extract.

### Cytotoxic activity

**Figure 3:**
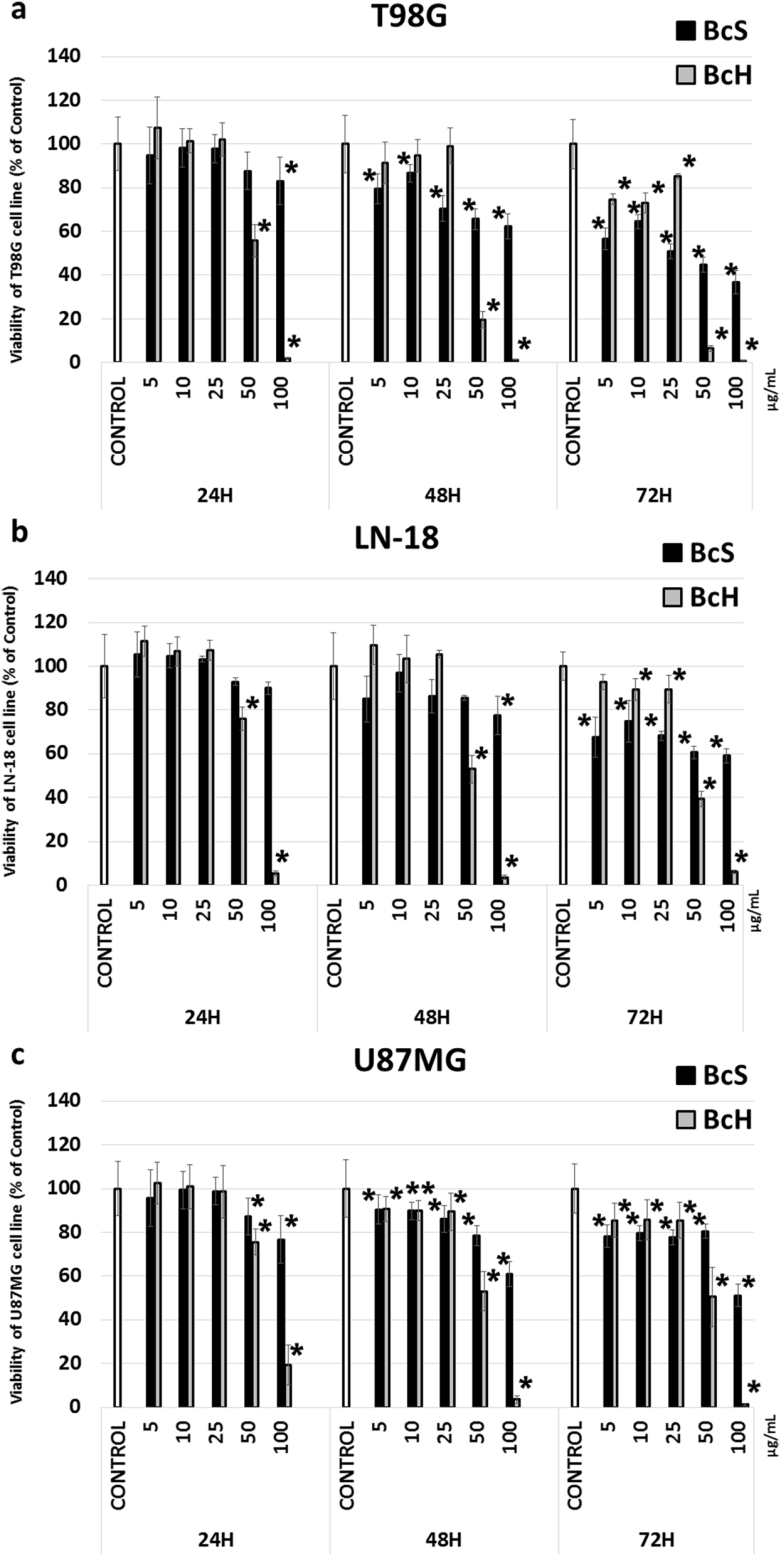
Viability of T98G (**a**), LN-18 (**b**) and U87MG (**c**) cells after 24, 48 and 72 h of incubation with BcH and BcS extracts (5, 10, 25, 50, 100 µg/mL). The results are presented as a percentage of control. All statistical analyses were performed using Student’s *t* test (significant changes: *p < 0.05 *vs* control).

**Figure 4:**
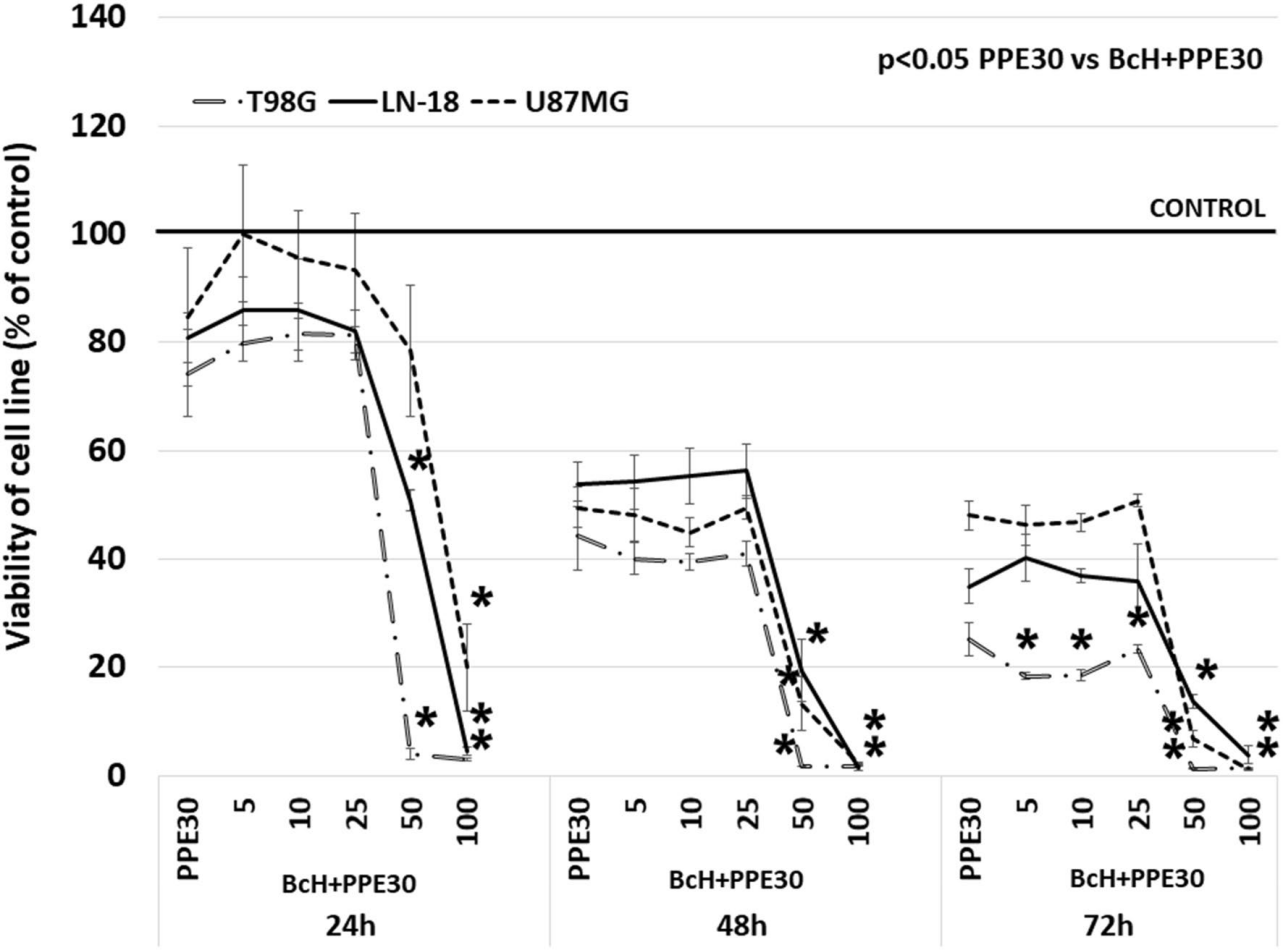
Viability of T98G, LN-18 and U87MG cells after 48 h of incubation with PPE30 (30 μg/mL) and BcH (5, 10, 25, 50, 100 µg/mL) combined with PPE30. The results are presented as a percentage of control. All statistical analyses were performed using Student’s *t* test (significant changes: *p < 0.05 PPE30 *vs* BcH + PPE30).

In this study, the cytotoxic effect of BcH, BcS (10–100 µg/mL) alone and BcH (10–100 µg/mL) in combination with PPE30 (30 µg/mL) was examined in T98G, LN-18 and U87MG cells. A time-dependent decrease was found in the cell viability of all tested glioma cell lines (Fig. 3a–c, Fig. 4). After 72 h of treatment with BcH, significant (*p* < 0.05) reductions in viability were observed at concentrations ≥ 5 µg/mL for T98G and U87MG cells and ≥ 10 µg/mL for LN-18 cells. The estimated IC_50_ values for T98G, U87MG and LN-18 were 35.9, 50.5 and 51.7 µg/mL, respectively. After 72 h of treatment with the BcS extract, significant (*p* < 0.05) reductions in viability were found at all concentrations for the studied glioma cell lines. The estimated IC_50_ values for T98G, U87MG and LN-18 cells were 40.9, 122.0 and 154.8 µg/mL, respectively. BcH extract was used for further study. Moreover, the quantitative determination of the composition of *B. monnieri* extracts showed a higher content of both, Bacoside A3 and Bacosaponin C in BcH than in BcS extract, which may explain the stronger cytotoxic effect of the BcH extract against glioblastoma multiforme cells. The cytotoxic effect of the combination of PPE and BcH extracts on T98G, U87MG and LN-18 cells was significantly higher (*p* < 0.05) than that of PPE used separately: after 24 h at concentrations of BcH ≥ 50 μg/mL for T98G and LN-18 and ≥ 100 μg/mL for U87MG; after 48 h at concentrations of BcH ≥ 50 μg/mL for all tested glioma cell lines and after 72 h at concentrations of BcH ≥ 5 μg/mL for T98G and ≥ 50 μg/mL for LN-18 and U87MG (Fig. 4).

### The effect on DNA synthesis in glioblastoma cell lines

**Figure 5:**
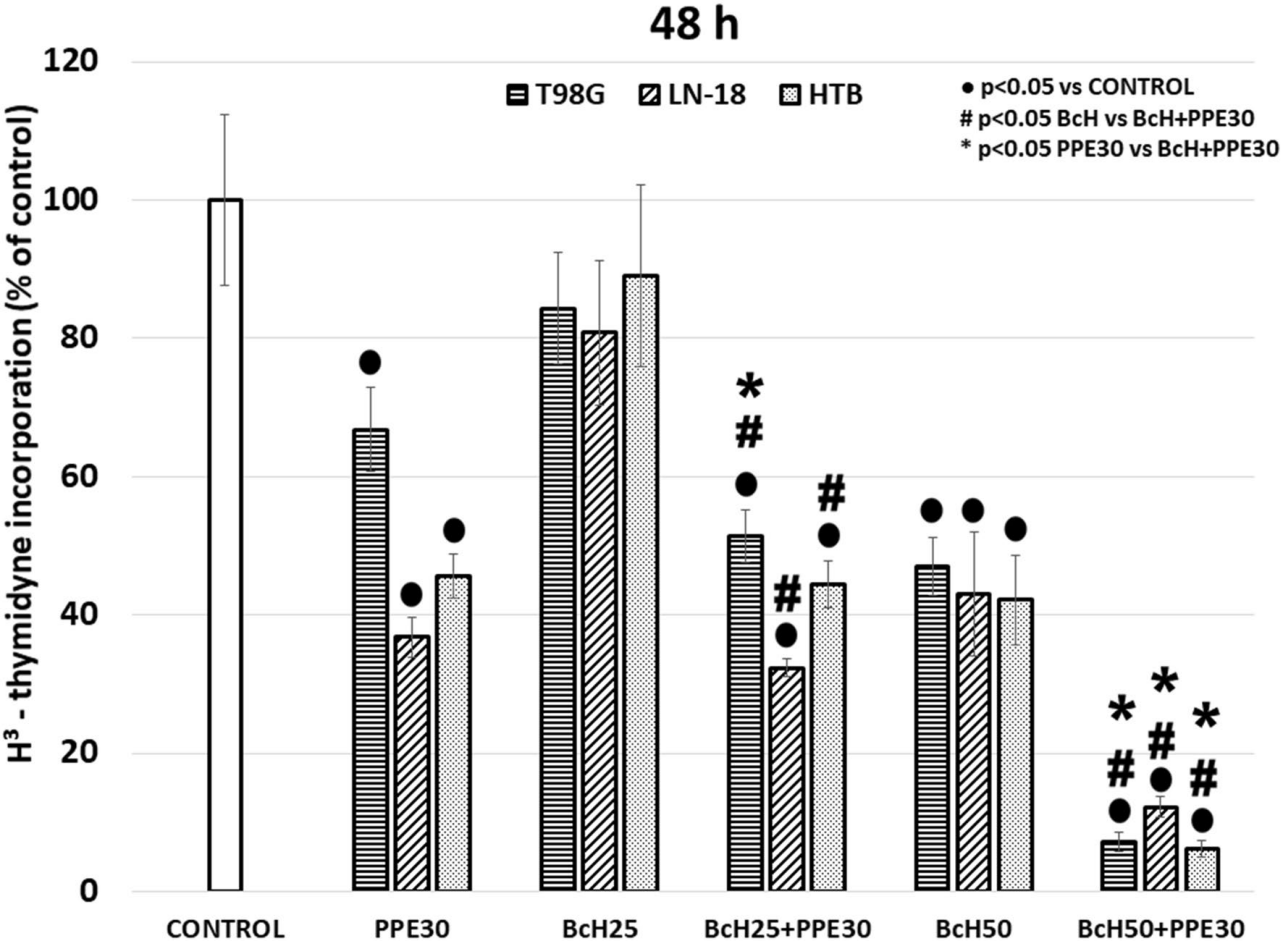
[^3^H]-thymidine incorporation on T98G, LN-18 and U87MG cells after 48 h of incubation with PPE30 (30 μg/mL), BcH (25, 50 μg/mL) and combination PPE30 with BcH. The results are presented as a percentage of control. All statistical analyses were performed using Student’s *t* test (significant changes: ●p < 0.05 *vs* control, # p < 0.05 BcH *vs* BcH + PPE30, *p < 0.05 PPE30 *vs* BcH + PPE30).

To examine the influence of the tested extracts and their combination on the DNA synthesis and proliferation of glioblastoma cells, the incorporation of [^3^H]-thymidine was evaluated. In this study, strong significant inhibition of DNA synthesis in T98G, LN-18, and U87MG cells was found after 48 h of incubation with PPE30 (66.8 ± 3.2, 36.8 ± 6.1, and 45.6 ± 2.9% of the control, respectively) and BcH50 (47.0 ± 6.5, 43.0 ± 4.2, and 42.2 ± 9.0% of the control, respectively) (Fig. 5). The combination of PPE30 with BcH50 significantly inhibited DNA synthesis in the tested cells (less than 13% of the control) (Fig. 5).

### Cell cycle

**Figure 6:**
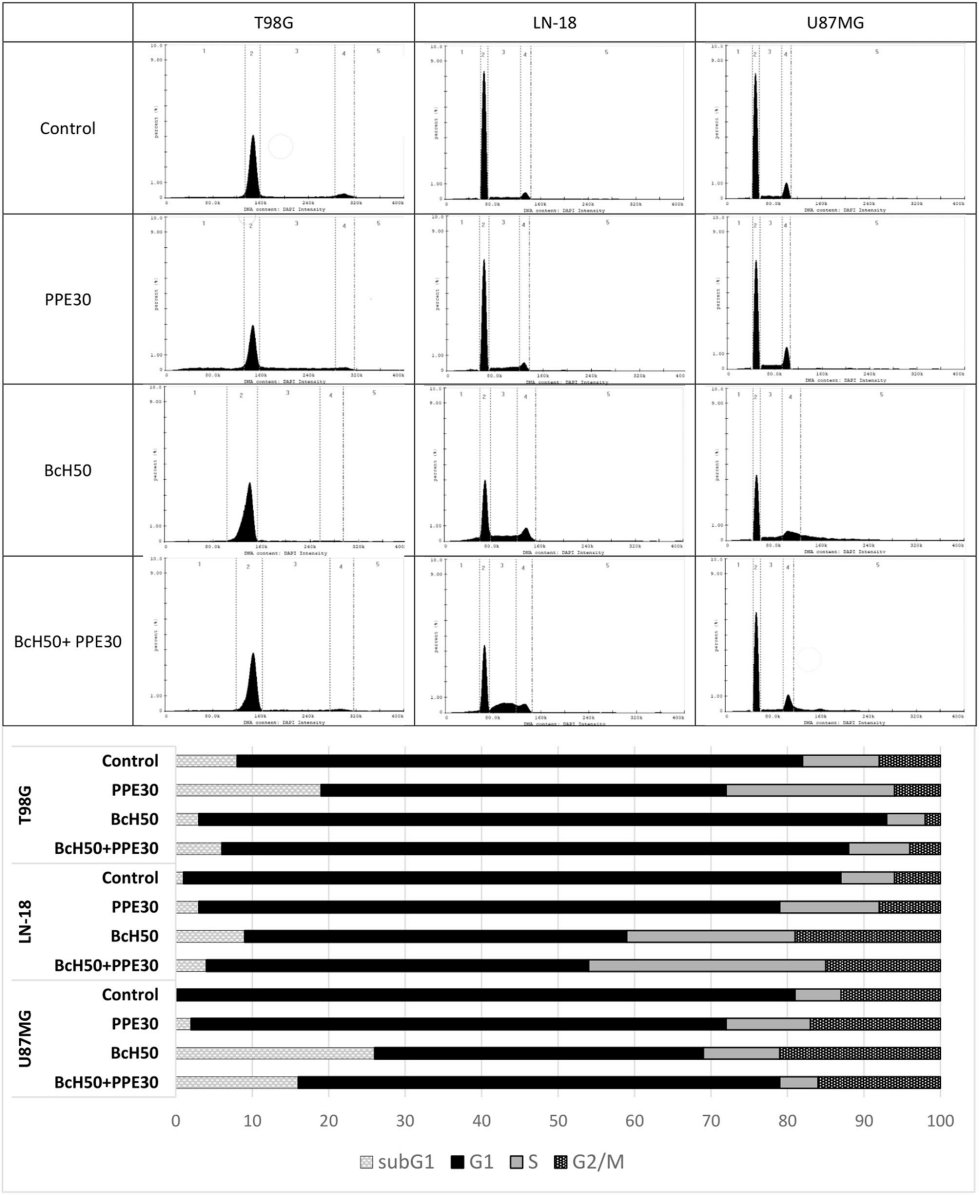
Cell cycle parameter analysis by flow cytometry. T98G, LN-18 and U87MG cells were incubated for 48 h with PPE30 (30 μg/mL), BcH50 (50 μg/mL) and their combination compared to controls. Both the histogram (**a**) and the bars (**b**) present the distribution of cells in subG1 (1), G1 (2), S (3) and G2/M (4) phases of the cell cycle.

The effect of PPE30, BcH50 and their combination on the cycle of T98G, LN-18 and U87MG cells after 48 h of incubation is presented in Fig. 6. Cell nuclei were stained, and the percentage of cells in specific cell cycle phases was calculated. Our data revealed that treatment with PPE30 extract caused increased cell cycle arrest (T98G cells: at subG1 phase by 11% and at S phase by 12%; LN-18 cells: at subG1 phase by 2% and at S phase by 6%; and U87MG cells: at subG1 phase by 2%, at S phase by 9% and at G2/M phase by 4% compared to the control). In fact, after treatment with BcH50, the percentage of cells in subG1 phase increased by approximately 8% (LN-18) and 26% (U87MG), the percentage of cells in G1 phase increased by approximately 16% (T98G), the percentage of cells in S phase increased by approximately 15% (LN-18) and 4% (U87MG), and the percentage of cells in G2/M phases increased by approximately 13% (LN-18) and 8% (U87MG) compared to the control. The combination of the studied extracts impacted the cell cycle by increasing the percentage of cells arrested at subG1 phase by approximately 3% (LN-18) and 16% (U87MG), the percentage of cells arrested at G1 phase by approximately 8% (T98G), the percentage of cells arrested at S phase by approximately 24% (LN-18), and the percentage of cells arrested at G2/M by approximately 9% (LN-18) and 3% (U87MG) compared to the control.

### Apoptosis quantification by Annexin V/PI staining

**Figure 7:**
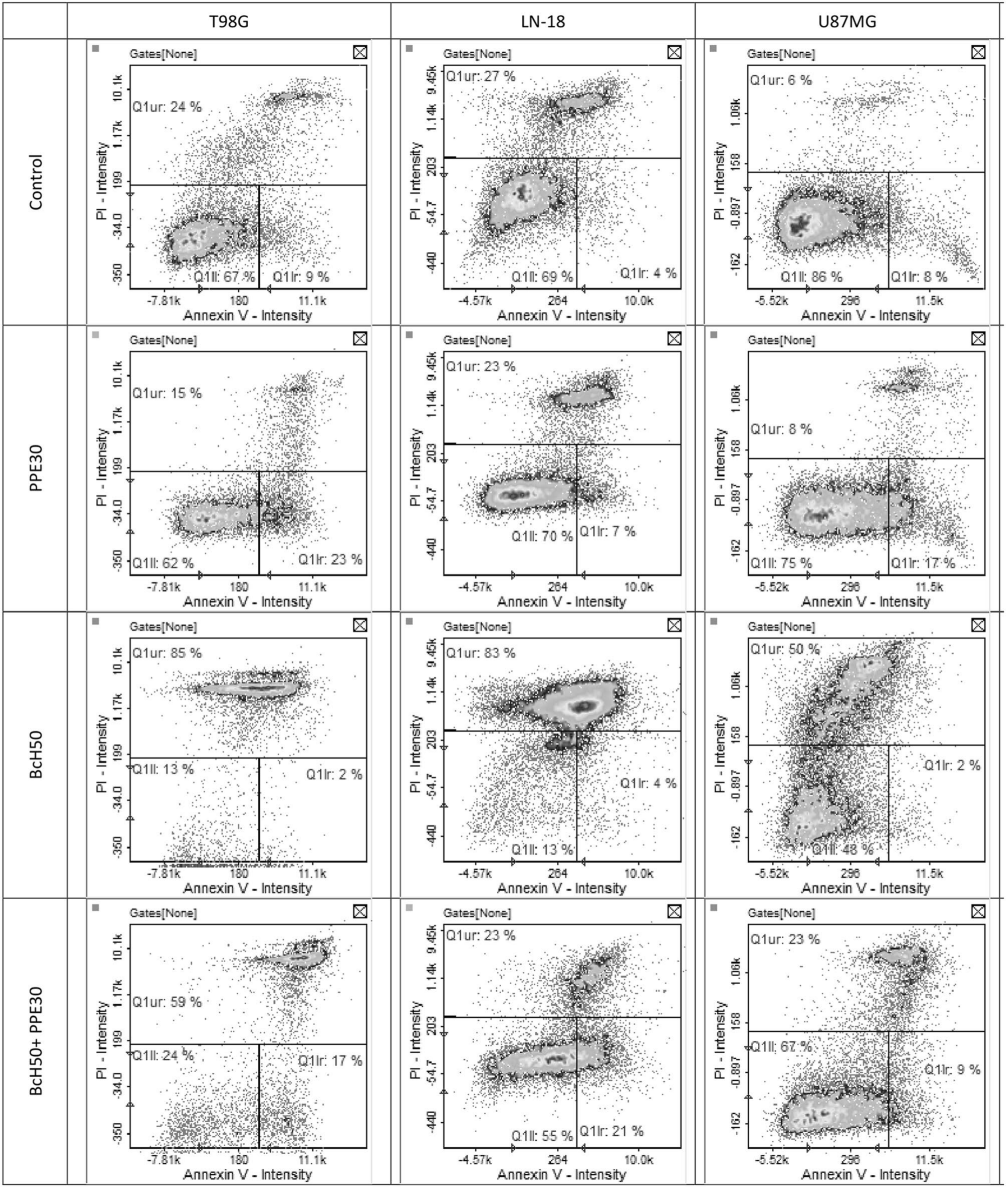
The quantitative assessment of T98G, LN-18 and U87MG cells apoptosis induced by PPE30 (30 μg/mL), BcH50 (50 μg/mL) and their combination using Annexin V/PI staining.

Annexin V and PI staining was used to assess the impact of treatment with the studied extracts and their combination on glioblastoma cell apoptosis (Fig. 7). Apoptosis quantification indicated that PPE30 caused an increase in the quantity of T98G, LN-18 and U87MG cells in early apoptosis (lower right quadrant) by approximately 14%, 3% and 9%, respectively, compared to the control. The effect of BcH50 on glioblastoma cells was based on the induction of late apoptosis and necrosis via an increased percentage of T98G, LN-18 and U87MG cells in the upper quadrant (approximately 61%, 56% and 44%, respectively). The combination of PPE30 and BcH50 exerts a partial effect on the induction of early apoptosis and late apoptosis/necrosis in the tested cell lines. The extract blend increased the ratio of T98G, LN-18 and U87MG cells in early apoptosis by approximately 8%, 17% and 1%, respectively. Moreover, the number of cells in late apoptosis/necrosis increased by 35% and 17% in the T98G and U87MG cell lines, respectively.

## Discussion

Glioblastoma multiforme (GBM) belongs to a group of the most lethal human cancers. Despite medical developments and the use of new cancer therapies and drugs, the mortality of GBM patients still remains high. Therefore, it is extremely important to continue to search for new compounds (also of natural origin) that could represent the origin of an anticancer drug or therapy. According to a European study of 956 patients carried out in 14 countries, as many as 50% of patients with brain tumors have used complementary and alternative medicine^4^. Thus, in this study, we aimed to evaluate the anticancer effect of Polish propolis extract (PPE) combined with *B. monnieri* extracts obtained from commercial products (BcH and BcS). According to the literature, the combined effect of PPE with bacopa has not been investigated thus far. This study provides, for the first time, evidence for the use of the combination in glioblastoma cell lines.

Propolis is a well-known product that contains many active substances that possess anticancer properties. However, its chemical composition and the number of active components might vary greatly depending on its location and other factors. In our study, the PPE sample was found to contain the flavonoids (Fig. 1) pinobanksin, pinobanksin 3-O-acetate, chrysin, and galangin and the phenolic acids *p*-coumaric, ferulic, caffeic and cinnamic acid esters. This compound is characteristic of propolis originating from the bud exudates of *Populus nigra*^6,28^. Other authors also proved the presence of these constituents in Polish propolis samples^6,29^. In addition, according to another study, the high amount of *p*-coumaric acid and ferulic acid as well as their esters (also found in our PPE sample) was very typical of the bud of *Populus tremula*^30,31^. The high content of phenolic compounds of PPE was confirmed in the TPC determination, where it was 137.19 ± 3.96 mg GAE/g. Other authors showed a similar or lower TPC in Polish propolis. The values ranged from 14.59 to 150.80 mg GAE/g and depended on the extraction method^32^. Due to the presence of a large amount of active substances, propolis has strong anticancer activity, which has been confirmed in many studies. Propolis from Brazil showed a strong inhibitory effect on cell growth in glioblastoma (U251 and U343) and fibroblast cell lines (MRC5) but had no effects on apoptosis, demonstrating a cytostatic effect^33^.

Apoptosis quantification indicated that PPE caused an increase in the quantity of T98G, LN-18 and U87MG cells in early apoptosis (approximately 14%, 3% and 9% compared to the control, respectively). On the other hand, according to Szliszka et al.^34^, propolis from Poland (50 μg/mL) caused an increase in the percentage of apoptotic cells (to 71.10%) in HeLa cells resistant to tumor necrosis factor-related apoptosis-inducing ligand (TRAIL). Furthermore, apigenin and CAPE may be the compounds responsible for this activity^11,35^. In our previous study, PPE was found to present cytotoxic properties and cooperate with temozolomide (TMZ), synergistically enhancing its growth-inhibiting activity against the glioblastoma U87MG cell line through the reduced activity of nuclear factor kappa-light-chain-enhancer of activated B cells (NF-κB)^14^.

The results of the cell cycle assay showed that treatment with PPE30 caused cell cycle arrest of T98G cells at the subG1 phase (11%) and S phase (12%), cell cycle arrest of LN-18 cells at the subG1 phase (2%) and S phase (6%), and cell cycle arrest of U87MG cells at the subG1 phase (2%), S phase (9%) and G2/M phase (4%) compared to the control. Studies on the effects of Lebanese propolis extracts on cell lines have also shown antiproliferative and cytotoxic effects, which are observed as an increase in the percentage of cells in the subG1 phase of the cell cycle. The percentage of Jurkat cells in subG1 increased after 24 h of incubation with both crude extract and hexane fractions. A significant increase in the percentage of cells in the subG1 phase was also observed in glioblastoma cells (U251) and breast adenocarcinoma (MDA-MB-231) after 24 h of exposure to the hexane fraction^36^.

The main goal of our research was to determine whether the combination of PPE and BcH and BcS would enhance the antitumor effect or, on the contrary, cause negative interactions and reverse effects. The reason that *B. monnieri* extract was used in our investigation was the significant amount of active substances, such as bacosides in this plant. A quality control for quantitative and qualitative analysis of BcH and BcS was proposed to analyze food supplements using liquid chromatography with coupled with mass spectrometer (LC–MS). The chemical composition of BcH and BcS was characterized by the dominance of the triterpene saponin complex. They are represented by jujubogenin derivatives, including bacoside A3 in both samples, as well as bacopasaponin A, E–G, and bacopaside III-IV. The second group of the same type of compounds were pseudojujubogenin derivatives such as bacopasaponin C, bacopaside II, and V. Two common flavones, luteolin and apigenin, as well as imino derivatives were also present in all samples of *B. monnieri*^37^.

Bacosides have a multidirectional pharmacological action. According to Ayurvedic medicine, the presence of bacoside A and bacoside B enhances memory. Bacosides inhibit lipoxygenase activity, scavenge free radicals and protect the neural cells of the prefrontal cortex, hippocampus, and striatum against cytotoxicity and DNA damage implicated in Alzheimer's disease. Furthermore, bacosides increase glutathione peroxidase, chelate iron and enhance nitric oxide-mediated cerebral vasodilation, leading to improvements in the total memory score^38^.

A prior study showed that *B. monnieri* plant extract and bacoside A enhance CaMK2A and activate its phosphorylation (pCaMK2A) in glioblastoma cells. Furthermore, pCaMK2A triggers an increase in high calcium release from the endoplasmic reticulum, causing tumor cell death. John et al.^39^ suggested that bacoside A has excellent brain availability through the oral route; hence, it should be proposed as a potential treatment for GBM. Other authors showed that *B. monnieri* (dichloromethane fraction) has anticancer potential against various human cancer cell lines—colon (HT29, Colo320, and Caco2), lung (A549), cervix (HeLa, SiHa), and breast (MCF-7, MDAMB-231) (IC_50_ 41.0–60.0 µg/mL after 72 h)—and in vivo in Ehrlich ascites carcinoma (EAC)-treated mice (at a dose of 40 mg/kg body weight). It has also been confirmed by in silico screening that the anticancer activity of *B. monnieri* may be due to the presence of bacosides and cucurbitacin^40^. Other studies have demonstrated that bacopaside II (a bacopa component) inhibits colon cancer cell growth at ≥ 20 µM for HT-29 cells and ≥ 15 µM for SW480, SW620 and HCT116 cells by inducing cell cycle arrest and apoptosis^41^.

In fact, after treatment with BcH50, the percentage of cells in subG1 increased by approximately 8% (LN-18) and 26% (U87MG), the percentage of cells in G1 phase increased by approximately 16% (T98G), the percentage of cells in S phase increased by approximately 15% (LN-18) and 4% (U87MG), and the percentage of cells in the G2/M phases increased by approximately 13% (LN-18) and 8% (U87MG) compared to the control. Smith et al.^41^ tested bacopaside II on SW620, SW480, HCT116 and HT-29 colon cancer cells. Their results showed that treatment with bacopaside II for SW620, SW480 and HCT116 cells resulted in an increase in the percentage of cells in G2/M phase and a concomitant decrease in G1 phase. In contrast, for HT-29, bacopaside II caused an increase in G0/G1 phase and a decrease in S phase, while a higher concentration of bacopaside II caused an increase in cells in G2/M phase and a concomitant decrease in cells in G0/G1 phase. These results suggest that cell death occurs after the use of this compound and is consistent with the observed reduction in cell growth^41^. The results of the research suggest that the main mechanism of *B. monnieri* is probably based on macropinocytosis, so the effect of these extracts is not observed upon examination of the cell cycle, despite a pronounced cytotoxic effect on glioblastoma cells. Some cancer lines after *B. monnieri* treatment have shown that the DNA fragmentation process is initiated, but the main mechanism of cell death is cell lysis, which occurs as a result of a combination of acute macropinocytotic overstress and anoiki necrosis, as demonstrated by John et al.^39^ in their investigation. Glioblastoma cells are vulnerable to high calcium-induced macropinocytotic hydrostatic stress. *B. monnieri* plant extract/bacoside A enhances calcium/calmodulin-dependent protein kinase type II subunit alpha (CaMK2A) and activates CaMK2A phosphorylation (pCaMK2A) in glioblastoma cells, and pCaMK2A triggers high calcium release from the endoplasmic reticulum, causing macropinocytotic and membrane hydrostatic stress-induced tumor cell death. Our studies revealed that the effect of BcH50 on glioblastoma cells was based on the induction of late apoptosis and necrosis via an increased percentage of T98G, LN-18 and U87MG cells (approximately 61%, 56% and 44%, respectively). Examination of bacopaside II in colon cancer cell lines (HT-29, SW480, SW620 and HCT116 cells) induced an increase in cell apoptosis. For HT-29 cells, bacopaside II induced a marked increase in the percentage of early and late apoptotic cells; in contrast, for SW480, SW620 and HCT116 cells, this marked increase occurred at a lower dose of bacopaside II^41^.

Our data showed a strong cooperative effect of propolis PPE30 and BcH50 on the studied human glioblastoma cell lines (T98G, LN-18, and U87MG cells). The inhibition of viability of the study glioma cells after incubation with the combination of PPE30 and BcH50 was significantly higher than that of these two extracts separately. The impact of PPE30 and BcH50 on DNA biosynthesis was examined by the [^3^H]-thymidine incorporation assay to confirm that inhibition of viability was caused by a reduction in proliferation capacity. We found that the combination of PPE30 and BcH50 was more powerful in inhibiting proliferation (up to 13%) than PPE30 or BcH50 alone (up to 67% and up to 47%, respectively).

The combination of PPE30 and BcH50 increased the percentage of cells arrested at subG1 phase by approximately 3% (LN-18) and 16% (U87MG); the percentage of cells arrested at G1 phase by approximately 8% (T98G); the percentage of cells arrested at S phase by approximately 24% (LN-18); and the percentage of cells arrested at G2/M phase by approximately 9% (LN-18) and 3% (U87MG) compared to the control. Moreover, the combination showed an effect on the induction of early apoptosis and late apoptosis/necrosis in the tested cell lines. The extract blend increased the ratio of T98G, LN-18 and U87MG cells in early apoptosis by approximately 8%, 17% and 1%, respectively. Moreover, the number of cells in late apoptosis/necrosis increased by 35% and 17% in the T98G and U87MG cell lines, respectively.

The mechanism of action of *B. monnieri* is probably related to the ability of bacoside A to bind to the CaMK2A enzyme, which induces phosphorylation and subsequently allows binding to ryanodine receptors located in endoplasmic reticulum membrane, which induce excessive calcium release. It leads to extensive fluid uptake via macropinocytosis and consequently causing swelling of the cell and trigger lysis. Propolis, as well as CAPE and chrysin, which are the main biologically active components of propolis, enhance the extrinsic pathway of apoptosis in cancer cells stimulated by TRAIL, tumour necrosis factor (TNF) or Fas receptors. CAPE, through activation of p38, mitogen-activated protein kinase (MAPK), c-Jun N-terminal kinase (JNK), and extracellular signal-regulated (ERK) kinases, confirms the involvement of the intrinsic pathway of apoptosis in the mechanism of anticancer activity. The suppression of anti-apoptotic proteins (IAP), cellular FLICE-inhibitory protein (c-FLIP), B-cell lymphoma 2 (Bcl-2), phosphatidylinositol 3-kinase (PI3K/Akt) kinase and inhibition of NF-kB activity by chrysin have also been reported^42^.

In summary, these results are the first to show that extracts from Polish propolis combined with *B. monnieri* have strong cytotoxic and antiproliferative effects on human glioblastoma cell lines. The activity may be associated with the high content of both polyphenolic compounds in propolis and bacosides in *B. monnieri.* These findings suggest that propolis in combination with *B. monnieri* shows promising anticancer activity for the treatment of glioblastoma cancer. However, further studies are still required.

## Materials and methods

### Chemicals and reagents

Dulbecco's modified Eagle’s medium (DMEM), Eagle’s minimal essential medium (MEM) with L-glutamine (292 mg/L), heat-inactivated fetal bovine serum (FBS), trypsin–EDTA, penicillin, and streptomycin were purchased from PAA Laboratories GmbH (Pasching, Austria); calcium-free phosphate buffered saline (PBS) was obtained from BIOMED (Lublin, Poland). Bis(trimethylsilyl)trifluoroacetamide (BSTFA) with the addition of 1% trimethylchlorosilane, alkane standard solutions C8-C20 and C21-C40, Folin–Ciocalteau reagent, methylthiazolyl diphenyl-tetrazolium bromide (MTT), dimethyl sulfoxide (DMSO), pyridine, sodium dodecyl sulfate solution, trichloroacetic acid, and (tris(hydroxymethyl) aminomethane hydrochloride) were obtained from Sigma-Aldrich (St. Louis, MO, USA). Ethanol at 95% was obtained from the AWW Group (Kalisz, Poland), and sodium carbonate (Na_2_CO_3_) was obtained from POCH (Gliwice, Poland). The scintillation cocktail was purchased from PerkinElmer (Boston, MA, USA). Methyl-^3^H thymidine was from MP Biomedicals, Inc. (Irvine, CA, USA). Bacoside A3 and bacoside A contain mixture bacoside A3, bacopaside II, bacopaside X, and bacopasaponin C were obtained from Sigma-Aldrich (St. Louis, MO, USA). Bacopasaponin C was purchased from Cayman Chemical Company (Ann Arbor, MI, USA). Luteolin and apigenin (purity > 96%) were isolated from inflorescences of *A*. *tomentosum*^43^. Acetonitrile Optima was purchased from Fisher Chemical (Thermo Fisher Scientific, Leicestershire, UK), and ultra-pure water (resistivity of 18.2 MΩ-cm) was obtained using the POLWATER DL3-100 system (Labopol, Kraków, Poland). Formic acid (Ph. Eur., Merck, Darmstadt, Germany) was used as the mobile phase modifier. Sodium sulfate buffer (0.05 M) (J.T. Backer, Deventer, Holland) prepared in ultra-pure water and adjusted to pH 2.3 with dilute sulphuric acid (P.P.H. Stanlab, Lublin, Poland) using HANNA Instruments pH meter (model edge, Woonsocket, RI, USA). All solutions were filtered through a 0.45 mm pore size membrane filter using a Macherey–Nagel syringe filter (Düren, Germany).

### Materials and extract preparation

*B. monnieri* extract (BcH) was stated to contain 44 mg of bacosides in one capsule (Hepatica, Niemce, Poland, batch no. 201605), and *B. monnieri* extract (BcS) BaCognize was stated to contain 30 mg of bacosides in one capsule (Swanson Health Products, Fargo, North Dakota, USA, batch no. SWH145). Both were purchased as food supplement. However, only the inner contents were analyzed. Concentrated ethanolic extract containing 67% Polish propolis was purchased from a manufacturer specializing in the production of apiculture product extracts from FINEX (Skawina, Poland, batch no. 150319). The solvent (95% ethanol) was evaporated under reduced pressure at 38 °C (Rotavapor R-3, Büchi, Flawil, Switzerland). The extract was lyophilized (Alpha 1–2 LD plus, Christ, Germany) before use and stored at -20 °C for further use. Before in vitro analysis, BcH, BcS and PPE were dissolved in DMSO and growth medium at a 1.0 mg/mL concentration as a stock solution. The final concentration of DMSO was 0.1%.

### GC–MS analysis of PPE

Five milligrams of PPE was diluted with 220 μL of pyridine and 80 μL of BSTFA with the addition of 1% trimethylchlorosilane. The reaction mixture was sealed and heated for 0.5 h at 60 °C to form trimethylsilyl (TMS) derivatives. GC–MS analysis of PPE was performed using a GC–MS Clarus 680 gas chromatograph with a Clarus 600 T MS mass selective detector (PerkinElmer, Walthman, MA, USA) equipped with an Elie-5 MS fused silica column (30 m, 0.25 mm i.d., 0.25 lm film thickness) with electronic pressure control and a split/splitless injector. The helium flow rate through the column was 1 mL/min in constant flow mode. The injector worked at 250 °C in split (1:50) mode. The initial column temperature was 40 °C, rising to 310 °C at 4 °C/min, and the higher temperature was maintained for 15 min. The MSD detector acquisition parameters were as follows: transfer line temperature 280 °C, MS source temperature 230 °C and MS quad temperature 150 °C. The EIMS spectrum was obtained at an ionization energy of 70 eV. The MSD was set to scan 41–600 a.m.u. After integration, the fraction of each component in the total ion current was calculated. Linear temperature programmed retention indices (I_T_) were the components, and both mass spectral data and the calculated retention indices were used. The identification was considered reliable if the results of a computer search in the mass spectra library (NIST 98; NIST/EPA/NIH Library of Electron Ionization Mass Spectra) were confirmed by the experimental I_T_^Exp^ values, i.e., if their deviation from the literature values I_T_^Lit^ (NIST Chemistry WebBook, 2013) did not exceed ± 7 u.i.

### LC–ESI–MS analysis of BcH and BcS

The quality analysis was carried out according to the method described by Nuengchamnong N. et al.^16^ with some modifications. The screening of BcH and BcS extracts was performed on a 1260 Infinity LC (Agilent, Santa Clara, CA, USA) consisting of a binary pump, a column oven and photo-diode array (PDA) detector combined with 6230 LC/TOF (Agilent, Santa Clara, CA, USA) mass spectrometer equipped with an electrospray ionization (AJS-ESI). The separation was performed using a ZORBAX Eclipse Plus C18 column (100 × 4.6 mm, 3.5 µm) (Agilent, Santa Clara, CA, USA). The mobile phase were water (A) and acetonitrile (B), both with the addition of 0.1% formic acid. The separation was achieved by a gradient of 0–8 min 35% B; at 8–10 min 35%–38% B; 10–14 min 38%–50% B; 14–22 min 50%–80% B; with next 5 min equilibration in the initial gradient. The flow rate was 0.6 mL/min and the column temperature was maintained at 35 ± 0.8 °C. The UV–Vis spectra was recorded from 190 to 540 nm with selective wavelength monitoring at 205 nm. The MS parameters used for the ionization source were set as follows: drying and sheath gas flow: 10 L/min; nebulizer: 30 psi; source temperature 350 °C; ion spray voltage 3500 V for the positive mode analysis. The data were collected in the 100–1200 m/z range and processing was performed using the Mass Hunter Qualitative analysis software.

### Quantitative assessment of saponins in BcH and BcS

The quantification was carried out according to the method described by Murthy et al.^44^ with some modifications. The contents of 3 capsules randomly selected from the package were mixed and homogenized. Fifty milligrams of the well-powdered sample was weighed and dissolved in a mixture of sodium sulfate buffer pH 2.3 and acetonitrile (50:50, *v/v*) assisting ultrasound (Sonic-5, Polsonic, Poland) for 10 min, filtered through a 0.45 mm filter, and made up to a final volume of 10 mL. The sample was prepared in triplicate. Two standards (bacoside A3 and bacopasaponin C, 1 mg/mL) in five levels were used to prepare the calibration curve in the range of 10–100 µg/mL. The calibration curve was calculated from the peak areas under the peak and presented as square equation regression (bacoside A3, R^2^ = 0.9999, y = 7.2171x + 10.604; bacopasaponin C, R^2^ = 0.9999, y = 8.2336x + 12.239). Quantity assessment was performed on the same 1260 Infinity LC system (Agilent, Santa Clara, CA, USA) equipped with ZORBAX SB-C18 column (250 × 4.6 mm, 5 µm) protected by a security column guard ZORBAX SB-C18 (12.5 × 4.6, 5 µm). The total analysis time was 75 min. The mobile phase was a mixture of sodium sulfate buffer (0.05 M) with pH 2.3 and acetonitrile in isocratic (68.5:38.5). Flow rate was set at 1 mL/min, selective wave length at 205 nm, and volume of injection was set at 20 µL.

### Total phenolic content (TPC) analysis

TPC was measured using the Folin–Ciocalteu colorimetric method. Twenty milligrams of BcH, BcS and PPE was dissolved in 10 mL of 95% ethanol and centrifuged (5 min, 200 rpm). Then, 0.25 mL of the supernatants was shaken for 5 min with 1.25 mL of 0.2 M Folin–Ciocalteau reagent, and after adding 1 mL of 7.5 g/L sodium carbonate (Na_2_CO_3_), the samples were incubated for 2 h at room temperature. The absorbances, in reference to the prepared blank, were read at 760 nm using a Cintra 3030 spectrophotometer (GBC Scientific Equipment, Braeside, Australia). The results were expressed as milligrams of gallic acid equivalent (GAE) per gram of dry extract. Assays were carried out in triplicate. Data are expressed as the mean ± SD.

### Cell culture

The study was performed using three human glioblastoma cell lines (T98G, LN-18, and U87MG) obtained from the American Type Culture Collection (ATCC, Rockville, MD, USA). The cells were cultured in a humidified incubator at 37 °C and 5% CO_2_ atmosphere in the growth medium – MEM (U98MG, T98G) or DMEM (LN-18) supplemented with 10% heat-inactivated FBS and 100 U/mL penicillin and 0.1 mg/mL streptomycin. Cell cultures were used between IX and XIV passages. Each experiment was performed in triplicate and repeated at least three times.

### Cytotoxicity assay

Cell viability was measured using the MTT assay as previously described for glioma cells^15^. The effect of BcH and BcS extracts and their combination with PPE30 (30 mg/mL) on human glioblastoma cell lines (T98G, LN-18, U87MG) was studied after 24 h, 48 h and 72 h of treatment. The cells were cultured in a humidified incubator at 37 °C in a 5% CO_2_ atmosphere in MEM (T98G, U87MG) and DMEM (LN-18) supplemented with 10% heat-inactivated FBS, 100 U/mL penicillin and 0.1 mg/mL streptomycin. The study dose of propolis was selected in our earlier experiments^14^. The cells were seeded into 96-well plates in a volume of 200 µL per well at a density of 1 × 10^5^ cells/mL and grown for 22 h at 37 °C in a humidified 5% CO_2_ incubator. The data are expressed as a percentage of the control (0.1% DMSO).

### DNA synthesis assay

[^3^H]-thymidine assays were performed to study DNA synthesis in the cells after treatment according to the protocol described in our previous study^15^. The cells were seeded (1.5 × 10^5^ cell/well) in 24-well plates in MEM or DMEM with 10% heat-inactivated FBS and exposed to the treatment medium containing DMSO (0.1%—control), BcH (25, 50 µg/mL), BcS (25, 50 µg/mL), PPE (30 µg/mL) and their combination. The cells were cultured for 44 h prior to adding 0.5 µCi of [^3^H]-thymidine per well. After 4 h of incubation, the medium was removed, and the cells were washed twice with cold 0.05 M tris(hydroxymethyl) aminomethane hydrochloride and 5% trichloroacetic acid, then lysed with 1% sodium dodecyl sulfate solution, scraped and transferred to a scintillation cocktail. The level of [^3^H]-thymidine incorporated in the newly synthesized DNA strand was assessed by a scintillation counter in relation to cells proliferating during the S phase of the cell cycle.

### Cell cycle assay

The impact of BcH50, PPE30 and the BcH50 + PPE30 mixture on the cell cycle was analyzed by the advanced image cytometer NucleoCounter NC-3000 (ChemoMetec, Lillerød, Denmark). The T98G, LN-18 and U87MG cells were seeded into 6-well plates at a density of 1 × 10^6^ cells per well. After 24 h of incubation, cells were treated with BcH50, PPE30 and BcH50 + PPE30 mixture. After 48 h of cell treatment, the test was performed according to the 2-step cell cycle assay protocol of the manufacturer (ChemoMetec, Lillerød, Denmark). Cellular fluorescence was measured at 365 nm and quantified. DNA content histograms were displayed on screen. Markers in the displayed histograms were used to demarcate cells in the different cell cycle stages. The results are presented as the percentages of the cells in different cell cycle phases: subG1, G1/G0, S or G2/M.

### Annexin V assay

Indications of early apoptosis were detected by initially staining the cells with Annexin V and propidium iodide (PI) solution followed by flow cytometry analysis using NucleoCounter NC-3000 (ChemoMetec, Lillerød, Denmark). The T98G, LN-18 and U87MG cells were seeded into 6-well plates at a density of 1 × 10^6^ cells per well, and after 24 h of incubation, the cells were treated with BcH50, PPE30 and BcH + PPE mixture. After 48 h of incubation with the studied agents, the assay was performed following the manufacturer’s protocol for the Annexin V assay (ChemoMetec, Lillerød, Denmark). The cells were harvested using trypsin/EDTA and centrifuged at 400 g for 5 min, and the supernatant was rejected. The cells were suspended in 100 μL of Annexin V binding buffer. Next, 2 μL of Annexin V-CF488A conjugate and 2 μL of solution containing Hoechst 33,342 were added. Then, samples were incubated at 37 °C for 15 min using a heating block (TS-100, bioSan, Rīga, Latvia). After incubation, the stained cells were centrifuged and washed twice with Annexin V binding buffer. Cell pellets were resuspended in 100 μL of Annexin V binding buffer supplemented with solution containing PI and analyzed immediately. After image acquisition and analysis scatter plots showing information about Annexin V-CF488A and propidium iodide fluorescence intensity, it was possible to determine the ratio of apoptotic and late apoptotic/necrotic cells. This test determined cell death via apoptotic or necrotic pathways. Normal living cells (lower left quadrant) were negatively stained for Annexin V-FITC and PI, and cells in early apoptosis (lower right quadrant) were positively stained with Annexin V-FITC and negatively with PI. Late apoptotic cells (upper quadrant) were positively stained with Annexin V-FITC and PI, and necrotic cells (upper quadrant) were negatively stained with Annexin V-FITC and positively with PI.

### Statistical analysis

All data were analyzed using Dell Inc. (2016) Dell Statistica (data analysis software system), version 13 (software.dell.com). The results were expressed as the mean ± SD and statistically compared to the control. Values were tested for normal distribution using the Shapiro–Wilk test. The differences between two groups were analyzed by Student’s *t* test. A *p* value < 0.05 was considered statistically significant.
